# Parental Knowledge, Attitudes, and Practices Towards Pediatric Ear Infections in the Makkah Region of Saudi Arabia: A Cross-Sectional Study

**DOI:** 10.7759/cureus.61990

**Published:** 2024-06-09

**Authors:** Nirmeen S Alnifaee, Rehaf F Althubaiti, Maha K Almatrafi, Turki A Alotaibi, Ghayad G Almjnouni, Ghadir K Sadakah, Amer M Khojah

**Affiliations:** 1 College of Medicine and Surgery, Umm Al-Qura University, Makkah, SAU; 2 Department of Pediatrics, College of Medicine and Surgery, Umm Al-Qura University, Makkah, SAU

**Keywords:** saudi arabia, mecca, makkah, practices, attitude, knowledge, pediatric, ear infections, otitis media

## Abstract

Background: Pediatric ear infections constitute a significant public health concern worldwide, adversely impacting children's health and well-being. Parents play a crucial role in prevention, ensuring timely healthcare access, and therefore minimizing potential complications. This study aims to assess parental knowledge, attitudes, and practices towards pediatric ear infections in Makkah region.

Methodology: A descriptive cross-sectional study was conducted among Saudi parents who were ≥18 years old and lived in Makkah region. Convenience sampling was used to recruit 319 participants through social media platforms; data were collected from June to September 2023 via an online self-administered questionnaire. The questionnaire assessed sociodemographic characteristics, along with knowledge, attitudes, and practices related to pediatric ear infections.

Results: A total of 319 parents were included in the study. The majority of the participants were female 228 (71.5%), and 208 (65.2%) had a university education level. The most common age groups were 18-30 years and 31-40 years. More than half of the participants (167, 52.4%) demonstrated adequate knowledge regarding pediatric ear infections. Positive attitudes and practices were reported by 183 (57.4%) and 285 (89.3%) of participants, respectively. Adequate knowledge was significantly higher among participants with younger ages (p<0.05). It was found that having a younger age (18-30 years) was an independent predictor of good knowledge (OR: 1.26 (1.96-3.65), p=0.009) and positive practice (OR: 1.53 (1.01-2.33), p=0.045).

Conclusion: We found that the majority of parents in Makkah region had a good level of knowledge regarding childhood ear infections, with an overall positive attitude and practice. The study revealed that younger parents had superior knowledge and younger age was an independent predictor for good knowledge and positive attitude.

## Introduction

Ear diseases pose a significant public health concern among pediatric patients in developing countries. If untreated, these conditions can cause significant social and psychological problems for both children and their families [1]. Among ear diseases, ear infections are the most common treatable cause of childhood morbidity [2]. Children are more prone to ear infections than adults due to a variety of factors, such as the anatomy of the middle ear and immature immune systems [3]. Otitis media (OM) is the leading cause of acute care visits and antibiotic prescriptions in children [4,5]. OM can be divided into acute otitis media (AOM), recurrent acute otitis media, otitis media with effusion (OME), and chronic suppurative otitis media (CSOM) [6]. These subcategories are based on the symptoms' onset, duration, and presence or absence of discharge [7]. 

Several studies show that 80% of children will have at least one episode of AOM by their third birthday and 40% will have six or more recurrences by the age of seven [8-10]. Furthermore, approximately 65-330 million individuals worldwide are afflicted with CSOM, with hearing impairment reported in 60% of these cases [11]. Hearing impairment in children has a negative impact on their speech development, learning ability, scholastic performance, quality of life, and social well-being [4].

Identifying and mitigating potential environmental risk factors linked to OM is crucial for decreasing its occurrence and minimizing complications [12]. Various risk factors for OM have been identified such as allergic rhinitis, adenoid hypertrophy, working mothers, pacifiers, and bottle feeding. However, among these, allergic rhinitis and adenoid hypertrophy emerge as the two most significant predictors of OM [13]. To prevent complications, pediatricians should be mindful of these risk factors and address them when managing a patient with OM. Parents' perceptions of OM risk may not always be accurate, leading to the underestimation of their child's true risk of developing an OM [14]. Parental education may help to reduce the likelihood of serious and life-threatening complications such as meningitis, brain abscess, mastoid abscess, facial nerve paralysis, and labyrinthitis [15,16]. Therefore, this study aimed to assess parental knowledge, attitudes, and practices towards pediatric ear infections in Makkah region of Saudi Arabia.

## Materials and methods

Study design

This is an observation descriptive cross-sectional study done in Makkah region of Saudi Arabia from June to September 2023. For data collection, the study used an online self-administrating questionnaire adapted from a previously published study [8]. The questionnaire was developed in English language and underwent translation into Arabic by a professional translator and was subsequently reviewed for consistency by investigators. Distribution of the questionnaire was facilitated through various social media platforms to reach the target population. The questionnaire took three to five minutes to complete and included a consent form, sociodemographic data, and knowledge, attitude, and practice sections.

Study subjects

Our inclusion criteria were Saudi parents aged ≥18 years living in Makkah region of Saudi Arabia. The exclusion criteria were illiterate and those who refused to participate in the study. A non-probability convenience sampling technique was used to achieve the desired sample; the sample size was calculated via OpenEpi Version 3.0 to determine the minimal sample size. The study was planned for a population of approximately 3787929 participants [17], and a minimum sample of 385 was required to achieve a 95% confidence level with a 5% error margin. 

Data analysis

Data were statistically analyzed using IBM SPSS Statistics for Windows, Version 26.0 (Released 2019; IBM Corp., Armonk, New York, United States). To investigate the association between the variables, the chi-squared test (χ2) was applied to qualitative data that was expressed as numbers and percentages. Multivariate logistic regression analysis was done to assess the risk factors (independent predictors) of adequate knowledge or positive attitude or practice related to ear infection. The odds ratio (OR) was calculated at a confidence interval (CI) of 95%. A p-value of less than 0.05 was regarded as statistically significant.

Study tool

Knowledge about ear infections was assessed across multiple domains such as etiology, manifestation, prevention, treatment, and complication of ear infections. Respondents were considered knowledgeable about etiology if they identified factors such as poor hygiene, wax, wetting the ears, foreign bodies, and/or microbes. Manifestations of ear infections included symptoms like ear discharge, hearing loss, fever, and pain, with respondents considered knowledgeable if they recognized three of the four symptoms. Prevention and treatment parameters were rated as "yes" or "no," with "yes" respondents considered knowledgeable. Respondents who reported hearing loss, extension into adjacent structures, disease persistence, or death were deemed knowledgeable. An average knowledge score was calculated based on respondents' proficiency across all knowledge domains.

Positive practices were defined by seeking medical treatment, whereas a positive attitude was inferred from the source of medical information. Respondents who reported "not being concerned," "no need for therapy," or "incurable" were classified as having a negative attitude. Furthermore, the choice for information seeking (community health worker, health professional) was classified as having a positive attitude. A sum average of each parameter's responses was computed to build a knowledge, attitude, and practice model.

Ethical considerations

Ethical approval for the study was obtained from the Umm Al-Qura University Institutional Research Board (IRB), Makkah, Saudi Arabia (approval number: HAPO-02-K-012-2023-10-1798). All data was used anonymously, and any identifiable information that would have disclosed a participant's identity was removed to safeguard participant confidentiality.

## Results

Of the studied 319 participants, 110 (34.5%) had an age ranging from 18 to 30 years, 228 (71.5%) were females, and 116 (36.4%) were residents of Makkah City. Most of them (284, 89%) were married, 208 (65.2%) had a diploma or university educational level, and 116 (36.4%) had a monthly income of 5000-10000 SR. The majority (239, 74.9%) had less than five children (Table 1).

**Table 1:** Distribution of the participants according to their demographic characters and children's data (n=319)

Variable	No. (%)
Age (years)	
18-30	110 (34.5)
31-40	89 (27.9)
41-50	79 (24.8)
>50	41 (12.9)
Caregivers' gender	
Female	228 (71.5)
Male	91 (28.5)
Residence	
Taif	30 (9.4)
Madinah	60 (18.8)
Jeddah	106 (33.2)
Makkah	116 (36.4)
Yanbo	5 (1.6)
Other	2 (0.6)
Marital status	
Widow	13 (4.1)
Married	284 (89)
Divorced	22 (6.9)
Educational level	
Secondary school or less	
Diploma or university	208 (65.2)
Postgraduate	26 (8.2)
Monthly income (SR)	
<5000	111 (34.8)
5000-10000	116 (36.4)
>10000	92 (28.8)
Children number	
<5	239 (74.9)
≥5	80 (25.1)

**Figure 1:**
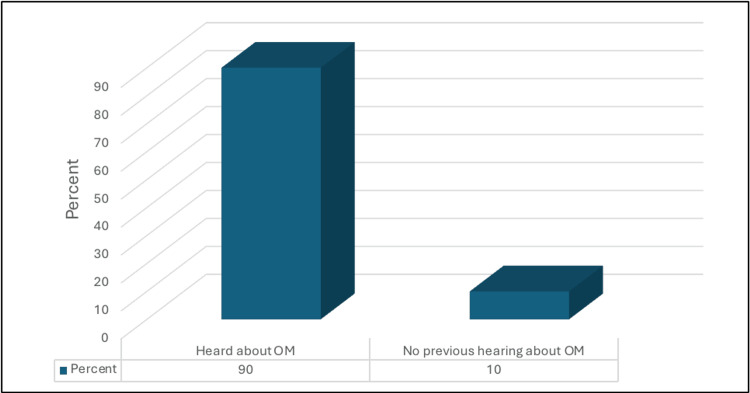
Percentage distribution of the participants according to previous hearing about OM (n=319) OM: otitis media

Of the participants, 90% had previously heard about OM (Figure 1).

Approximately 122 (38%) of the participants knew ≥3 symptoms of ear infection. One hundred and eighty-nine (59.2%) of the participants were knowledgeable of the causes of ear infections, and 231 (72.4%) were knowledgeable about prevention measures. Moreover, a significant percentage of them (302, 94.7%) were knowledgeable about treatment, while only 153 (48%) were knowledgeable about potential complications of ear infections. In general, more than half (167, 52.4%) of the participants demonstrated adequate knowledge about ear infections. Regarding participants' attitudes and practices, 183 (57.4%) displayed a positive attitude towards ear infections, and 285 (89.3%) had positive practice (Table 2).

**Table 2:** Participants' knowledge, attitudes, and practices related to ear infection (n=319)

Variable	No. (%)
Knowledge about the manifestation of ear infections	
1 symptom	63 (19.7)
2 symptoms	80 (25.1)
≥3 symptoms	122 (38.2)
I don't know	54 (16.9)
Knowledgeable about the etiology of ear infections	
Yes	189 (59.2)
No	130 (40.8)
Knowledgeable about prevention	
Yes	231 (72.4)
No	88 (27.6)
Knowledgeable about treatment	
Yes	302 (94.7)
No	17 (5.3)
Knowledgeable about the consequences of infections	
Yes	153 (48)
No	166 (52)
General knowledge about ear infection	
Adequate knowledge	167 (52.4)
Inadequate knowledge	152 (47.6)
Attitude towards ear infection	
Positive attitude	183 (57.4)
Negative attitude	136 (42.6)
Practice related to ear infection	
Positive practice	285 (89.3)
Negative practice	34 (10.7)

**Figure 2:**
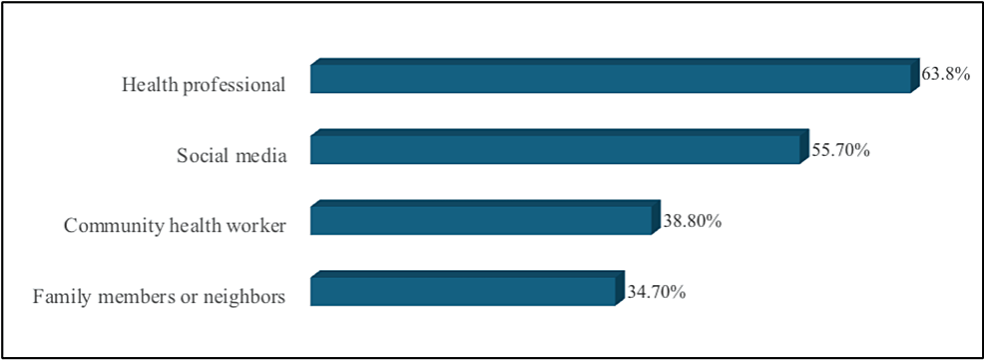
Percentage distribution of information sources on health matters among participants

Figure 2 illustrates that healthcare professionals were the most common (68.3%) source of information on health matters among participants.

Table 3 shows the relationship between the participants' knowledge level and their attitudes and practices. These relationships were not statistically significant.

**Table 3:** Relationship between knowledge level about ear infections and attitude and practice level (n=319)

Variable	Knowledge level		χ2	P-value
	Inadequate no. (%)	Adequate no. (%)		
Attitude level				
Positive attitude	114 (60.3)	69 (53.5)	1.46	0.226
Negative attitude	75 (39.7)	60 (46.5)		
Practice level				
Positive practice	165 (87.3)	121 (93.8)	3.57	0.059
Negative practice	24 (12.7)	8 (6.2)		

Furthermore, adequate knowledge about ear infections was significantly higher among participants in the younger age group (18-30 years) compared to other age groups (p<0.05) (Table 4).

**Table 4:** Relationship between knowledge level about ear infections and participants' demographics and children number (n=319)

Variable	Knowledge level		χ2	P-value
	Inadequate no. (%)	Adequate no. (%)		
Age (years)				
18-30	48 (36.9)	62 (32.8)	8.83	0.032
31-40	45 (34.6)	44 (23.3)		
41-50	24 (18.5)	55 (29.1)		
>50	13 (10)	28 (14.8)		
Caregivers' gender				
Female	99 (76.2)	129 (68.3)	235	0.125
Male	321 (23.8)	60 (31.7)		
Marital status				
Widow	3 (2.3)	10 (5.3)	3.42	0.181
Married	115 (88.5)	169 (89.4)		
Divorced	12 (9.2)	10 (53)		
Educational level				
Secondary school or less	30 (23.1)	55 (29.1)	1.57	0456
Diploma or university	88 (67.7)	120 (63.5)		
Postgraduate	12 (9.2)	14 (7.4)		
Monthly income (SR)				
<5000	47 (36.2)	64 (33.9)	0.6	0.74
5000-10000	44 (33.8)	72 (38.1)		
>10000	39 (30)	53 (28)		
Children number				
<5	100 (76.9)	139 (73.5)	0.46	0.494
≥5	30 (23.1)	50 (26.5)		

Multivariate logistic regression analysis was done to assess the risk factors (independent predictors) of adequate knowledge or positive attitude or practice related to ear infection among studied participants. It was found that having a younger age (18-30 years) was an independent predictor of good knowledge (OR: 1.26 (1.96-3.65), p=0.009) or positive practice (OR: 1.53 (1.01-2.33), p=0.045) related to ear infection, while none of the studied variables was an independent predictor of positive attitude (p≥0.05) (Table 5).

**Table 5:** Multivariate logistic regression analysis of risk factors of adequate knowledge or positive attitude or practice related to ear infection

Variable	B	Wald	P-value	OR (CI: 95%)
Knowledge about ear infection				
Age (years)	1.23	2.77	0.009	1.26 (1.96-3.65)
Caregivers' gender	0.22	0.55	0.456	1.24 (0.69-2.24)
Marital status	0.7	3.61	0.057	0.49 (0.24-1.02)
Educational level	0.08	0.14	0.708	0.91 (0.57-1.46)
Monthly income (SR)	0.09	0.31	0.576	0.9 (0.64-1.27)
Children number	0.12	0.16	0.684	0.88 (0.48-1.6)
Attitude towards ear infection				
Age (years)	0.03	0.07	0.78	1.03 (0.79-1.35)
Caregivers' gender	0.2	0.5	0.477	1.23 (0.69-2.17)
Marital status	0.13	0.15	0.693	0.87 (0.43-1.73)
Educational level	0.4	2.94	0.816	1.5 (0.94 -2.4)
Monthly income (SR)	0.1	0.4	0.525	0.89 (0.64-1.25)
Children number	0.15	0.28	0.894	0.85 (0.47-1.53)
Practice related to ear infection				
Age (years)	0.42	4.02	0.045	1.53 (1.01-2.33)
Caregivers' gender	0.11	0.06	0.793	0.12 (0.46-1.72)
Marital status	0.45	0.74	0.237	1.57 (0.56-1.42)
Educational level	0.11	0.09	0.762	1.12 (0.53-2.35)
Monthly income (SR)	0.09	0.12	0.723	0.9 (0.52-1.55)
Children number	0.47	0.97	0.423	0.62 (0.24-1.59)

## Discussion

OM is a common ear infection primarily affecting children, particularly those under seven years of age [18]. There are different types of OM, including secretory OM, AOM, and chronic OM, each caused by various infectious agents [19]. The involvement of parents is crucial in managing their children's infections. Given the pivotal role of parents in managing their children's infections, their understanding of OM's causes and risk factors can play a significant role in preventing infection. Furthermore, parents' attitudes towards seeking medical care have been found to have a considerable impact on reducing complications associated with OM, such as brain abscess, facial palsy, and meningitis [15]. In this study, we assess parental knowledge, attitudes, and practices towards pediatric ear infections in Makkah region of Saudi Arabia.

Regarding parental knowledge, our results showed most of the participants (167, 52.4%) demonstrated sufficient knowledge regarding ear infections. This finding is consistent with other similar studies conducted in Riyadh, the capital and most populated city in Saudi Arabia [20,21]. However, this contrasts with studies conducted in Jazan, which found that most of the participants exhibited inadequate knowledge of OM [22,23]. This disparity suggests a variability in parental knowledge of ear infections based on the region and population studied. Of note, the study conducted in Jazan, a smaller province in the southern area of Saudi Arabia, included over 60% of participants residing in rural areas, potentially contributing to the lower levels of knowledge observed [22]. This underscores the importance of tailored educational interventions to bridge these knowledge gaps and ensure comprehensive understanding among all demographic groups.

In terms of awareness, 90% of the participants reported previous knowledge of OM. Two hundred and two (63.3%) of the parents recognized at least two symptoms of OM which is consistent with a prior study conducted in Jazan [23]. This relatively high level of awareness is promising and could potentially lead to the early detection and treatment of OM. 

Regarding the knowledge of ear infection causes, prevention, consequences, and therapy, our study revealed varied levels of knowledge among participants. While 189 (59.2%) were knowledgeable about ear infection causes and 153 (48%) about consequences, a majority (231, 72.4%) were aware of prevention methods, and nearly all (302, 94.7%) were knowledgeable about therapy. In comparison with studies conducted in Navi-Mumbai and Riyadh, our study showed a lower knowledge level about the causes and consequences of OM. For example, these studies reported that 77% and 82% of the parents knew about one or more causes of OM and around 95% of parents were knowledgeable about the infection consequences. On the other hand, parents' prevention knowledge in our study was similar to the other two studies which found that about 70% of the parents were knowledgeable about prevention in the two studies, respectively [20,24].

For attitude and practice, a substantial proportion of participants demonstrated positive attitudes (183, 57.4%) and practices (285, 89.3%) towards ear infections. Notably, positive attitudes and knowledge were independently associated with the 18-30 age group, possibly due to easier access to updated resources among younger parents. This finding contrasts with previous studies where age did not significantly associate with knowledge or attitude towards ear infections [20]. Health professionals and community health workers emerged as primary sources of health education, indicating a reliance of the participants on medical expertise and guidance. These findings are consistent with prior research conducted in similar settings [20,23,25].

There were some limitations to this study. First, the data was obtained using self-reported questionnaires, which means there is a possibility of inaccurate information due to reporting bias and misunderstanding. Additionally, the questionnaire may not have comprehensively covered all the aspects of ear infections. Future studies would benefit from exploring a broader range of factors that could influence parental practices related to pediatric ear infections. Furthermore, it is important to note that the results of this study may not be generalizable to the entire population of Saudi Arabia due to the cultural differences across various regions and populations within the country.

## Conclusions

We found that most parents had an adequate level of knowledge regarding childhood ear infections, with an overall positive attitude and practice. The study revealed that younger parents demonstrated superior knowledge, with younger age being an independent predictor for both good knowledge and positive attitude.
